# Placental growth factor for the prognosis of women with preeclampsia (fullPIERS model extension): context matters

**DOI:** 10.1186/s12884-020-03332-w

**Published:** 2020-11-05

**Authors:** U. Vivian Ukah, Beth A. Payne, Jennifer A. Hutcheon, Lucy C. Chappell, Paul T. Seed, Frances Inez Conti-Ramsden, J. Mark Ansermino, Laura A. Magee, Peter von Dadelszen, for the fullPIERS Group

**Affiliations:** 1 Department of Epidemiology, Biostatistics and Occupational Health, McGill University, Montreal, QC, H3A 1A2 Canada; 2 BC Children’s Hospital Research Institute, Vancouver, BC Canada; 3 School of Population and Public Health, University of British Columbia, Vancouver, BC Canada; 4 Department of Obstetrics and Gynaecology, University of British Columbia, Vancouver, BC Canada; 5 Department of Women and Children’s Health, School of Life Course Sciences, King’s College London, London, UK; 6 Department of Anaesthesiology, Pharmacology and Therapeutics, University of British Columbia, Vancouver, BC Canada

**Keywords:** Preeclampsia, Prediction, Placental growth factor, Prognosis, Hypertension, Model extension, Maternal health

## Abstract

**Background:**

The fullPIERS risk prediction model was developed to identify which women admitted with confirmed diagnosis of preeclampsia are at highest risk of developing serious maternal complications. The model discriminates well between women who develop (vs. those who do not) adverse maternal outcomes. It has been externally validated in several populations. We assessed whether placental growth factor (PlGF), a biomarker associated with preeclampsia risk, adds incremental value to the fullPIERS model.

**Methods:**

Using a cohort of women admitted into tertiary hospitals in well-resourced settings (the USA and Canada), between May 2010 to February 2012, we evaluated the incremental value of PlGF added to fullPIERS for prediction of adverse maternal outcomes within 48 h after admission with confirmed preeclampsia. The discriminatory performance of PlGF and the fullPIERS model were assessed in this cohort using the area under the receiver’s operating characteristic curve (AUROC) while the extended model (fullPIERS +PlGF) was assessed based on net reclassification index (NRI) and integrated discrimination improvement (IDI) performances.

**Results:**

In a cohort of 541 women delivered shortly (< 1 week) after presentation, 8.1% experienced an adverse maternal outcome within 48 h of admission. Prediction of adverse maternal outcomes was not improved by addition of PlGF to fullPIERS (NRI: -8.7, IDI − 0.06). Discriminatory performance (AUROC) was 0.67 [95%CI: 0.59–0.75] for fullPIERS only and 0.67 [95%CI: 0.58–0.76]) for fullPIERS extended with PlGF, a performance worse than previously documented in fullPIERS external validation studies (AUROC > 0.75).

**Conclusions:**

While fullPIERS model performance may have been affected by differences in healthcare context between this study cohort and the model development and validation cohorts, future studies are required to confirm whether PlGF adds incremental benefit to the fullPIERS model for prediction of adverse maternal outcomes in preeclampsia in settings where expectant management is practiced.

## Background

Pre-eclampsia and other hypertensive disorders of pregnancy complicate approximately 10% of pregnancies and contribute considerably to maternal, fetal, and newborn morbidity and mortality, worldwide [1]. Preeclampsia, defined broadly as hypertension with symptoms or signs of end-organ compromise, can lead to severe maternal complications (e.g., eclampsia, stroke, and liver dysfunction) and/or fetal complications (e.g., stillbirth and preterm delivery) [2, 3]. Early identification of women at high risk of these complications can guide care and prevent delays in treatment in order to prevent poor outcomes [2].

The fullPIERS risk prediction model was developed to facilitate early identification of women admitted with confirmed preeclampsia who are at greatest risk of developing severe maternal complications (e.g. eclampsia and stroke, see Appendix **S****1** for full list of outcomes) within 48 h of admission [4]. The published model (equation presented in Table 1) showed good discriminatory performance with an area under the receiver-operating characteristic curve (AUROC) of 0.88 (95% confidence interval [CI], 0.84–0.92) upon internal validation and AUROC 0.81 (95% CI 0.75–0.86) upon external validation [5]. Thus, the model can aid in risk stratification, to allow for corticosteroid administration, transfer to higher care facilities, and plan for delivery for high-risk women.

**Table 1:** Original versus Extended fullPIERS Logistic Regression Equations for the prediction of adverse maternal outcomes from pre-eclampsia

Original fullPIERS Logistic Regression Equation	Extended fullPIERS Logistic Regression Equation
logit (pi) = 2·68 + (− 5·41 × 10^− 2^; gestational age at eligibility) + 1·23(chest pain or dyspnea)+ (− 2·71 × 10^− 2^; creatinine) + (2·07 × 10^− 1^; platelets) + (4·00 × 10^− 5^; platelets^2^)+ (1·01 × 10^− 2^; aspartate transaminase (AST))+ (− 3·05 × 10^− 6^; AST^2^) + (2·50 × 10^− 4^; creatinine×platelet) + (− 6·99 × 10^− 5^; platelet×AST) + (− 2·56 × 10^− 3^; platelet×SpO_2_)	logit (pi) = − 1.34 + (0.25 × fullPIERS model linear predictor) + (− 0.01 × PlGF) **Odds ratio** (adverse maternal outcome) = 0.26 + (1.28 × fullPIERS model linear predictor [95% CI 1.23–1.33]) + (0.99 × PlGF [95% CI 0.98–0.99])

Since the development of fullPIERS, new biomarkers have been introduced that could aid the identification of adverse outcomes in preeclampsia. One such biomarker is placental growth factor (PlGF), an angiogenic factor found in the maternal circulation [6, 7]. Plasma concentrations of PlGF are decreased in pregnancies complicated by preeclampsia compared with uncomplicated pregnancies [6–10]. Several studies have tested the diagnostic ability of PlGF for women with suspected preeclampsia [6, 11–13]; the PELICAN study reported 96 and 98% sensitivity and negative predictive value, respectively, using PlGF <5th percentile, to predict confirmed preeclampsia and subsequent delivery within 14 days among women presenting with suspected preeclampsia before 35 + 0 weeks of gestation [11]. Similarly, in a cluster-randomized trial, PlGF < 100 pg/ml identified women (95 and 98% sensitivity and negative predictive value, respectively), with suspected preeclampsia who delivered within 14 days with confirmed pre-eclampsia [12]; these findings were consistent in the PreEclampsia Triage by Rapid Assay (PETRA) trial with a sensitivity of 92.5% and specificity of 63.8% [14]. However, fewer studies have aimed to investigate the prognostic value of PlGF in women with confirmed preeclampsia. In women with suspected preeclampsia, high discriminatory performance or strong likelihood ratios (LRs) have been demonstrated for PlGF for combined adverse maternal and fetal outcomes (usually the need for delivery with pre-eclampsia within 7–14 days) [10, 14–18]; however there are limited studies reporting on solely maternal outcomes [18–22].

Based on these reports, we evaluated whether addition of PlGF to fullPIERS could improve prediction of adverse maternal outcomes in women with confirmed preeclampsia.

## Methods

This manuscript was prepared using the Transparent reporting of a multivariable prediction model for individual prognosis or diagnosis (TRIPOD) reporting guidelines for prediction models (**Table S****2**) [23].

### Study cohort

For this study, we used the PETRA data which included women that had PlGF measurements during admission [8, 14]. The original data originally consisted of a prospective cohort of 1217 women presenting with suspected symptoms and signs of preeclampsia with measurable PlGF values, at 24 maternity units in the United States of America (USA; *N* = 23) and Canada (*N* = 1) (November 2010 to January 2012) [14]. Women from the PETRA [8, 14] cohort who had confirmed preeclampsia were included in the study.

### PlGF measurement

Maternal plasma samples were collected from eligible women between 20 and 35 weeks’ gestation, during the study recruitment. Samples were tested for PlGF using the Triage PlGF Test (Quidel Inc., San Diego, USA) according to the manufacturer’s instructions. We restricted our analyses to women with PlGF measured during hospital admission for confirmed preeclampsia (or at most 14 days before admission), to reduce the potential effect of changing PlGF concentration over time in the analyses and to be consistent with the other fullPIERS variable measurements [4, 5].

### Definition of pre-eclampsia and outcomes

To be consistent with the fullPIERS model development study [5], preeclampsia was defined as hypertension plus one of proteinuria, hyperuricemia, or as HELLP (Hemolysis, Elevated Liver enzyme, Low Platelet) syndrome. A composite adverse maternal outcome was defined as the occurrence of maternal death or any maternal morbidity as determined by Delphi consensus (see online [https://pre-empt.cfri.ca/monitoring/fullpiers] and Appendix S1).

### Statistical analyses

The distribution of participant characteristics included in this study were compared with those of women in the original fullPIERS development cohort, using Chi-squared and Mann–Whitney U tests (*p*-value of < 0.05) for categorical and continuous variables, respectively. Univariable analyses were also carried out comparing characteristics of women in our study cohort with those in previous studies, according to low PlGF (< 100 pg/mL) and normal PlGF (≥100 pg/mL) [18].

### Model extension with PlGF

PlGF measurements were converted to percentiles based on the reference range by gestational age interval [24]. Before inclusion in the fullPIERS model, we assessed the univariable discriminatory performance of PlGF. Using the most abnormal values of test results of the model predictors within 48 h of admission, a linear predictor variable was calculated for all the women in our study cohort using the fullPIERS model equation (1).

A logistic regression model was fitted with two variables: (i) the linear predictor and (ii) converted PlGF percentiles [7]. Hence, a new intercept and slope were estimated for the fullPIERS model as well as a regression coefficient for PlGF (Table 1). The extended model was then used to calculate the predicted probabilities of experiencing an adverse outcome for each woman and its performance was evaluated.

### Prediction performance measures

The ability of PlGF to predict adverse maternal outcomes and the performance of the original fullPIERS model in the data were evaluated based on discrimination capacity. Discriminative ability was assessed using the AUROC and was interpreted using the following pre-specified criteria: non-informative (AUROC ≤0.5), poor discrimination (0.5 < AUROC ≤0.7), good discrimination (AUROC > 0.7) [25]. In addition to discrimination, the extended model performance was evaluated based on the net reclassification index (NRI) and integrated discrimination improvement (IDI) to assess the incremental value of PlGF compared with the performance of the original model [26–28].

NRI gives a summary of the overall improvement of sensitivity and specificity associated with addition of PlGF to fullPIERS. NRI integrates the “upward movement” (improved reclassification) and the “downward movement” (worse reclassification) for the women with adverse outcomes in the predicted risk groups with the reverse movements for the women without adverse outcomes [29, 30]. The sum of the differences between the two movements for the women with and without adverse outcomes is the NRI.

IDI is the difference in the discrimination slope between the original model and the extended model. The discrimination slope is calculated as the difference between the average predicted probabilities of the women with and without adverse maternal outcomes. This measurement also incorporates the NRI across all possible cut-offs.

All analyses were done in R version 3.5.1 (R Foundation for Statistical Computing, Vienna, Austria).

## Results

**Fig. 1:**
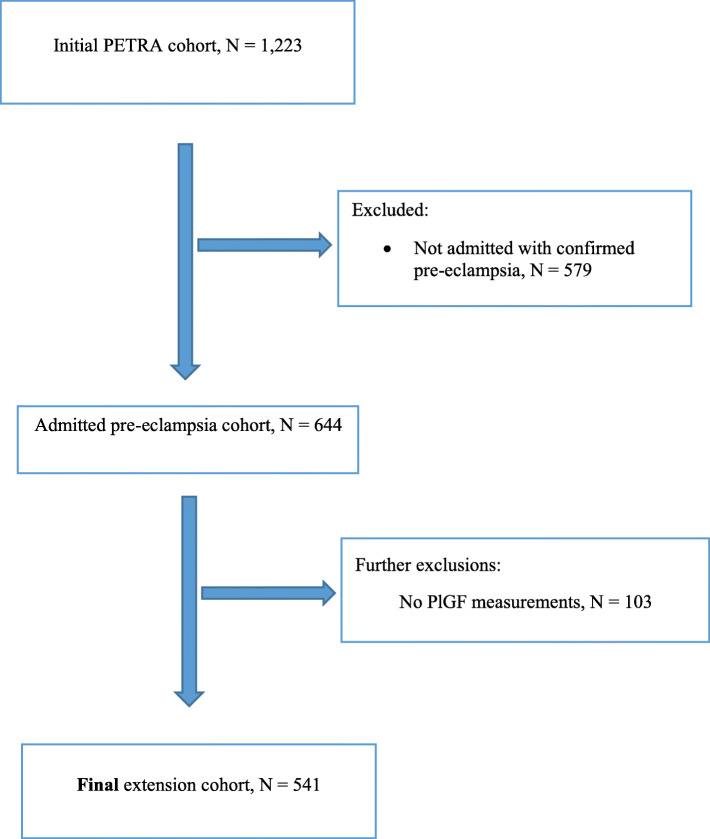
Flow showing identification of study population

The extension cohort included only a subset of women who met our inclusion criteria (*N* = 541), as previously described (Fig. 1).

### Demographics

The women in the extension cohort (compared with those in the original fullPIERS development cohort) were different in a number of ways (Table 2). In general, women admitted with preeclampsia in the original fullPIERS cohort were known to be managed expectantly, whereas those admitted in the extension cohort settings (mostly in the USA) were more likely to be delivered; this was also evident in the shorter admission-to-delivery interval. On average, the women in the extension cohort were younger with lower blood pressure measurements and presenting with earlier-onset of preeclampsia than the women in the fullPIERS cohort. They were also more likely to be multiparous, smokers, and receive magnesium sulfate; have a shorter admission-to-delivery interval with early-onset preeclampsia (gestational age [GA] < 34 weeks); and have babies with lower birth weight, compared with the fullPIERS cohort. There were no meaningful differences in the proportions of multifetal pregnancies or treatment with antihypertensive medication. Adverse maternal outcomes occurred in 8.1% of women within 48 h of admission, 9.6% within 7 days of admission, and 10.5% at any time during admission.

**Table 2:** Maternal characteristics for the fullPIERS Development cohort vs Extension cohort, (n (%) or median (interquartile range))

Characteristics	fullPIERS cohort (development) (2023 women)	Extension cohort (541 women)	***P*** value
**DEMOGRAPHICS & PREGNANCY CHARACTERISTICS**			
**Maternal age at EDD (year)**	31 [27, 36]	30 [24, 34]	< 0.001
**Parity ≥ 1**	581 (28·7%)	219 (40.4%)	< 0.001
**Gestational age at eligibility (week)**	36 [33, 38·3]	33.0 [29.7, 35.9]	< 0.001
**Gestational age at eligibility < 34 weeks, N**	636 (31.4%)	307 (56.7%)	< 0.001
**Multiple pregnancy**	192 (9·5%)	43 (8.0%)	0.2468
**Smoking in this pregnancy**	249 (12·3%)	118 (21.8%)	< 0.001
**INTERVENTIONS DURING ADMISSION**			
**Corticosteroids, GA onset < 34**	440/636 (69.2%)	128/307 (41.7%)	< 0.001
**Antihypertensive therapy**	1381 (68·3%)	384 (71.0%)	0.054
**MgSO**_**4**_	690 (34·1%)	410 (75.8%)	< 0.001
**PREGNANCY OUTCOMES**			
**Admission-To-Delivery Interval, < 34**^**+ 0**^ **Weeks (Days), mean (SD)**	10.9 (11)	5.0 (8)	< 0.001
**Gestational age at delivery (week), median (IQR)**	36.9 [34·1, 38·6]	33.9 [30.5, 36.4]	< 0.001
**Stillbirth**	20 (1.0%)	10 (1.9%)	0.2489
**Neonatal death**	26 (1·3%)	12 (2.2%)	0.0989
**ADVERSE MATERNAL OUTCOME (N women)**			
**Within 48 h**	106 (5.2%)	44 (8.1%)	0.023
**Within 7 days**	203 (10.0%)	52 (9.6%)	0.067
**At anytime**	261 (12.9%)	57 (10.5%)	0.243

Abbreviations: EDD – Estimated date of delivery, GA – Gestational age, MgSO_4_ – Magnesium sulphate, SD – standard deviation, IQR – interquartile range

PlGF was classified as low (< 100 pg/ml) in 485 women (89.6%) (Table 3). Low maternal PlGF concentrations were more likely among women with earlier onset of preeclampsia, higher blood pressure, and babies with lower birth weight. Low PlGF was present in almost all women who went on to experience adverse maternal (*N* = 53/57, 93.0%) at any time during admission.

**Table 3:** Maternal characteristics for Normal vs Low PlGF values in the Extension data, (n (%) or median (interquartile range))

Characteristics	Low PlGF (< 100 pg/ml), n (%) or median (IQR), (***n*** = 485)	Normal PlGF (≥100 pg/ml), n (%) or median (IQR), (***n*** = 56)
**DEMOGRAPHICS & PREGNANCY CHARATERISTICS**		
**Maternal age at EDD (year)**	30 [24, 34]	29 [26, 34]
**Parity ≥ 1**	192 (39.6%)	27 (48.2%)
**Gestational age at eligibility (week)**	32.6 [29.6, 35.7]	35.2 [33.4, 36.8]
**Gestational age at eligibility < 35 weeks, N**	329 (67.8%)	26 (46.4%)
**Multiple pregnancy**	40 (8.2%)	3 (5.4%)
**Smoking in this pregnancy**	101 (20.8%)	17 (30.4%)
**CLINICAL MEASURES**		
**Systolic BP (mm Hg)**	144 [135, 155]	136 [126, 150]
**Diastolic BP (mm Hg)**	86 [78, 94]	80 [74, 91]
**Uric acid**	375 [315, 435]	297 [256, 351]
**Lowest platelet count (× 10**^**9**^ **per L)**	199 [154, 243]	226 [160, 251]
**Highest AST/ALT (U/L)**	24 [19, 37]	18 [16, 24]
**Creatinine**	62 [53, 71]	53 [44, 62]
**INTERVENTIONS DURING ADMISSION**		
**Corticosteroids, GA onset < 35**	127/329 (38.6%)	7/26 (26.9%)
**Antihypertensive therapy**	348 (71.8%)	36 (64.3%)
**MgSO**_**4**_	376 (77.5%)	34 (60.7%)
**PREGNANCY OUTCOMES**		
**Admission-To-Delivery Interval (Days)**	2 [1, 4]	2 [1, 7]
**Gestational age at delivery (week)**	33.3 [30·1, 36.3]	36.1 [34.7, 37.3]
**Stillbirth**	10 (2.1%)	0
**Neonatal death**	12 (2.5%)	0
**MATERNAL OUTCOME (N women)**		
**Within 48 h**	41 (8.5%)	3 (5.4%)
**Within 7 days**	49 (10.1%)	3 (5.4%)
**At anytime**	53 (10.9%)	4 (7.1%)
**PlGF measurement-To-adverse maternal outcome Interval (at any time, Days)**	3 [2, 6] Mean = 4	3 [1, 5] Mean = 2

Abbreviations: AST (aspartate aminotransferase), BP (blood pressure), EDD (estimated date of delivery), MgSO_4_ (magnesium sulphate)

### Prediction of adverse maternal outcomes after adding PlGF to fullPIERS

**Fig. 2:**
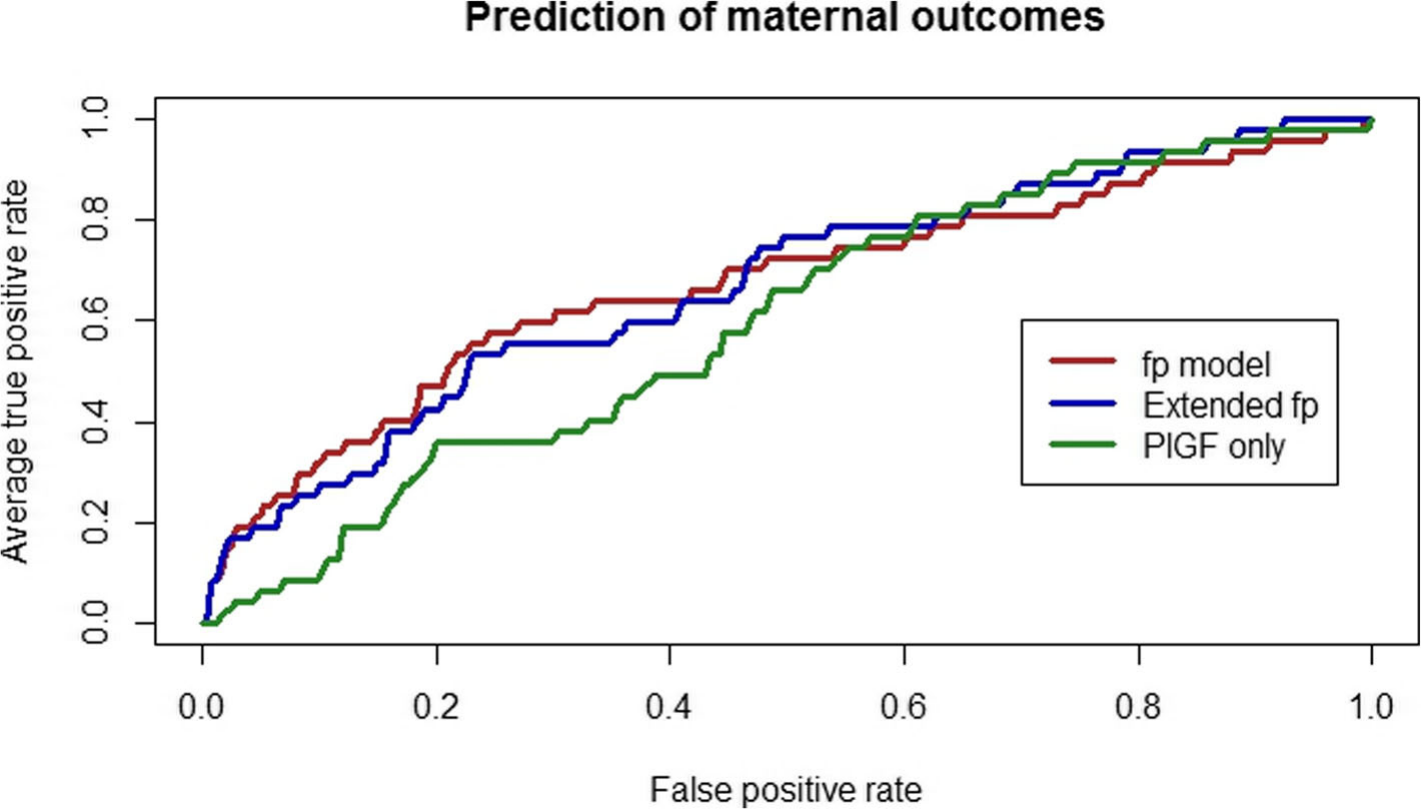
Prediction of adverse maternal outcomes using (i) only PlGF (0.60 (95% CI 0.52–0.68)) (ii) original fullPIERS (fp) model (0.67 (95% CI 0.58–0.76)) and (iii) the extended fullPIERS model (0.67 (95% CI 0.59–0.75)) in the extension cohort

In our analyses, the original fullPIERS model (AUROC 0.67 [95% CI 0.58–0.76]) and PlGF alone (AUROC 0.60 [95% CI 0.52–0.67]) showed poor discriminatory performance for prediction of adverse maternal outcomes within 48 h of admission in the extension cohort **(**Fig. 2**)**. Addition of PlGF to fullPIERS neither altered the odds of an adverse maternal outcome (OR 0.98 [95% CI 0.99–1.00]) for every percentile increase in PlGF (Table 1), nor discriminatory performance (AUROC 0.67 [95% CI 0.59–0.75]) **(**Fig. 2**)**.

**Fig. 3:**
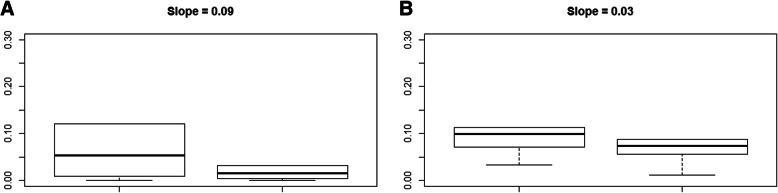
Discrimination slopes for (**a**) Original model (without PlGF) and (**b**) Extended model with PlGF (IDI = − 0.06) in the extension cohort

With addition of PlGF to fullPIERS, using a threshold of ≥10% for the calculated predicted probabilities, there were eight upward movements of women with adverse maternal outcomes into the high-risk category and two fewer cases without adverse maternal outcomes in the highest-risk category compared with the classification using the original model **(**Table 4**)**. The overall improvement in specificity was 11.9%, with a decrease of 18.4% in sensitivity using the extended model. Thus, the NRI was − 8.7, indicating no overall improvement in reclassification of risks. The discrimination slope for the extended model (fullPIERS plus PlGF) was 0.03, compared with 0.09 for the original model **(**Fig. 3**)**. The IDI was − 0.06, also indicating no meaningful improvement in discrimination.

**Table 4:** Reclassification Table for Extended Model with PlGF (fullPIERS plus PlGF)

fullPIERS Model without PlGF			
Predicted probability	Women with events	Women without events	Total
**0 to 9%**	37	475	**512**
**≥ 10%**	7	22	**29**
**Total**	**44**	**497**	**541**
**fullPIERS model with PlGF**			
**Predicted probability**	**Women with events**	**Women without events**	Total
**0 to 9%**	29	416	**445**
**≥ 10%**	15	81	**96**
**Total**	**44**	**497**	**541**

**Net reclassification index (NRI) calculation**

*Original fullPIERS model*

Sensitivity = 37/44*100 = 84.1%; Specificity = 22/497*100 = 4.4%

*fullPIERS model + PlGF (extended fullPIERS model)*

Sensitivity = 29/44*100 = 65.9%; Specificity =81/497*100 = 16.3%

Improved sensitivity = 65.9–84.3% = −18.4%; Improved specificity = 16.3–4.4% = 11.9%

NRI = Improved sensitivity + Improved specificity = − 18.4% + 9.7% = **−8.7%**

## Discussion

### Principal findings

In our study comprised of women with confirmed preeclampsia from tertiary hospitals in well-resourced settings, prediction of adverse maternal outcomes was not improved by addition of PlGF to fullPIERS. Of note is that fullPIERS performed poorly in this extension cohort compared with other studies where the AUROC was > 0.75 [4, 5, 31].

### Results compared to other studies

In the original fullPIERS model development cohort, the model performed well in the prediction of adverse maternal outcomes (AUROC 0.88, 95% CI 0.84–0.92). Similarly, in methodologically rigorous and robustly conducted temporal and external validation studies [4, 5], the model also performed well, (AUROC of 0.82 (95% CI 0.76–0.87) for temporal and 0.81 (95% CI 0.75–0.86) for external validation). In another study assessing the model in women with early-onset of pre-eclampsia, the discriminatory performance of the model was AUROC of 0.80 (95% 0.75–0.86). However, in this study, the model performance was lower, even upon the addition of PlGF. These findings are similar to an abstract assessing the fullPIERS model in a cohort of women admitted with pre-eclampsia in the United States, which reported a lower performance (AUROC: 0.68, 95% CI 0.60–0.76) for adverse maternal outcomes [32]. The fullPIERS was developed and externally validated in settings where expectant management was the practice. We hypothesize that this difference in model performance may be due to differences in pre-eclampsia management between the original fullPIERS setting and the other hospital settings in the USA. Further research to test this hypothesis would be valuable.

In contrast with our study, the PARROT trial reported lower incidence of adverse maternal outcomes in women with suspected preeclampsia (rather than confirmed preeclampsia as studied here), when PlGF values were revealed to the clinician, compared with the concealed group [12]. However, in the PETRA study [14] (from which our study extension cohort was derived), clinicians were masked to PlGF values and, therefore, could not have been influenced by the results. Although the PARROT trial results suggest that clinicians might positively respond to low PlGF possibly by increasing surveillance for women potentially at higher risk of adverse outcomes, it did not assess the prognostic value of PlGF for adverse maternal outcomes in women with preeclampsia.

### Strengths

To our knowledge, this is the first study to assess the addition of PlGF to fullPIERS for prediction of adverse maternal outcome in women with confirmed preeclampsia. Our use of a composite outcome that warrants consistent clinical action makes the results of our study robust and clinically relevant. This evaluation of an externally-validated model provides benchmark performance estimates against which comparisons can be made for different locations and upon addition of new biomarkers.

### Limitations

The main limitation of our study was having limited power due to a relatively small sample size and number of adverse outcomes. Based on our small sample size, we did not re-estimate the fullPIERS model coefficients when extending the model with PlGF as this would lead to significant overfitting of the model in the data set [33]. However, this may have been necessary if the addition of PlGF were to change the predictive coefficients of the other variables in the model. Second, we may have underestimated the relationship between PlGF and adverse outcome because some PlGF measurement results were carried forward (by no more than 14 days), rather than ideally have been measured within 48 h of admission. Third, practice varied in our study settings; expectant care (pregnancy prolongation) was less frequently practised in the extension cohort setting, so the natural history of disease was truncated by expedited delivery. In contrast, in the fullPIERS model development cohort, expectant care was the norm, resulting in a longer admission to delivery interval (mean of 11 days, compared with 5 days in the extension cohort – Table 2).

### Clinical implications

Despite our study limitations outlined above, there are possible explanations for the poor predictive performance for adverse maternal outcomes observed in our study. PlGF is a marker of placental dysfunction; therefore, this angiogenic factor may be more reflective of the initiation of preeclampsia (i.e. from suspected to established pre-eclampsia), rather than the progression or disease severity after the diagnosis of preeclampsia. Thus, PlGF appears to be less useful as a prognostic marker for women with confirmed pre-eclampsia in settings where less expectant management is practised.

Of note, almost 90% of included women had low PlGF suggesting a higher risk cohort. Such a case-mix is likely to reduce prediction performance, as more population homogeneity is associated with lower discrimination [34]. However, the high proportion of low PlGF values supports in the role of this angiogenic marker as a risk marker of preeclampsia development; women with low PlGF were more likely to have higher blood pressure measurements and experience worse neonatal outcomes.

## Conclusion

In our study, the addition of PlGF did not improve the performance of the fullPIERS model in predicting adverse maternal outcomes in the extension cohort. Given the poor performance of fullPIERS for prediction of adverse maternal outcomes in this study compared with previous studies, we speculate that our findings may relate to differences in case-mix and/or context between this cohort and the original fullPIERS model development cohort. These data suggest that fullPIERS may not be predictive of maternal outcome in settings where expedited delivery for preeclampsia is the standard of care. Given the paucity of relevant datasets for these analyses, future work is needed to evaluate the incremental benefit of adding PlGF or other biomarkers e.g. sFLT-1 to fullPIERS, a model based on routinely collected maternal history, physical examination, and laboratory variables, especially in healthcare settings with expectant management.

## Supplementary information

**Additional file 1: S1.** List and definitions of PIERS maternal adverse Outcomes. **S2.** TRIPOD checklist for reporting prediction model development and validation studies.

## Data Availability

The data that support the findings of this study are available from Quidel triage Inc. (https://www.quidel.com/immunoassays/triage-test-kits) but restrictions apply to the availability of these data, which were used under license for the current study, and so are not publicly available. Data are however available from the authors upon reasonable request and with permission of Quidel triage Inc.

## Abbreviations

PlGF: Placental growth factor
CI: Confidence interval
AUROC: Area under the receiver’s operating characteristic curve
NRI: Net reclassification index
IDI: Integrated discrimination improvement
PETRA: PreEclampsia Triage by Rapid Assay
GA: Gestational age
AST: Aspartate transaminase
sFLT-1: Soluble fms-like tyrosine kinase-1
