# Supplementary material for: Placental growth factor for the prognosis of women with preeclampsia (fullPIERS model extension): context matters
Source: BMC Pregnancy Childbirth. 2020 Nov 5;20:668. doi: 10.1186/s12884-020-03332-w (PMC7643272; doi:10.1186/s12884-020-03332-w)
Supplement: Supplementary file 1 — Additional file 1: S1. List and definitions of PIERS maternal adverse Outcomes. S2. TRIPOD checklist for reporting prediction model development and validation studies. [file 12884_2020_3332_MOESM1_ESM.docx]

**S1.** List and definitions of PIERS Maternal Adverse Outcomes

| **Outcome** | **Definition** |
| --- | --- |
|  | |
| Maternal Mortality | Maternal death occurring within six weeks of pregnancy or if later, attributable to complications of pre-eclampsia |
| Hepatic dysfunction | International Normalized Ratio (INR) >1.2 in the absence if Disseminated intravascular coagulation (DIC) or treatment of Warfarin (DIC is defined as having both: abnormal bleeding and consumptive coagulopathy (i.e., low platelets, abnormal peripheral blood film, or one or more of the following: increased INR, increased PTT, low fibrinogen, of increased fibrin degradation products that are outside normal non-pregnancy ranges)) |
| Hepatic hematoma or rupture | Blood collection under the hepatic capsule as confirmed by ultrasound or laparotomy |
| Glasgow Coma Scale (GCS) < 13 | Based on Glasgow coma scale (GCS) scoring system: Teasdale G, Jennet B. Assessment of coma and impaired consciousness: a practical scale. *Lancet* 1974; **2**:81-83 |
| Stroke | Acute neurological event with deficits lasting longer than 48 hours |
| Cortical Blindness | Loss of visual acuity in the presence of intact papillary response to light |
| Reversible Ischaemic Neurologic Deficit (RIND) | Cerebral ischaemia lasting longer than 24 hrs but less than 48 hours revealed through clinical examination |
| Retinal detachment | Separation of the inner layers of the retina from the underlying retinal pigment epithelium (RPE, choroid) and is diagnosed by opthamological exam |
| Acute renal insufficiency | For women with an underlying history of renal disease: defined as creatinine >200 uM; for patients with no underlying renal disease: defined as creatinine >150 uM |
| Dialysis | Including haemodialysis and peritoneal dialysis |
| Platelet count < 50,000 without blood transfusion | Measurement of platelet count recorded as less than 50,000 without patient being given a blood transfusion |
| Transfusion of blood products | Includes transfusion of any units of blood products: fresh frozen plasma (FFP), platelets, red blood cells (RBCs), cryoprecipitate (cryo) or whole blood |
| Positive inotropic support | The use of vasopressors to maintain a systolic blood pressure (sBP) > 90 mmHg or Mean Arterial pressure > 70 mmHg |
| Myocardial ischaemia/infarction | Electrocardiogram (ECG) changes (ST segment elevation or depression) without enzyme changes AND/OR any one of the following: 1)Development of new pathologic Q waves on serial ECGs. The patient may or may not remember previous symptoms. Biochemical markers of myocardial necrosis may have normalized, depending on the length of time that has passed since the infarct developed. 2) Pathological findings of an acute, healed or healing MI 3) Typical rise and gradual fall (troponin) or more rapid rise and fall (CK-MB) of biochemical markers of myocardial necrosis with at least one of the following: a) ischaemic symptoms; b) development of pathologic Q waves on the ECG; c) ECG changes indicative of ischaemia (ST segment elevation or depression); or d) coronary artery intervention (e.g., coronary angioplasty) |
| Require >50% oxygen for greater than one hour | Oxygen given at greater than 50% concentration based on local criteria for longer than 1 hour |
| Intubation other than for Caesarean section | Intubation may be by ventilation, Electrical impedance tomography (EIT) or Continuous positive airway pressure/power (CPAP) |
| Pulmonary Oedema | Clinical diagnosis with x-ray confirmation or requirement of diuretic treatment and Oxygen saturation (SaO_2_) <95% |

**S2.** TRIPOD Checklist for reporting Prediction model development and validation studies

| **Section/Topic** | **Item** |  | **Checklist Item** | **Page** |
| --- | --- | --- | --- | --- |
| **Title and abstract** | | | | |
| Title | 1 | D;V | Identify the study as developing and/or validating a multivariable prediction model, the target population, and the outcome to be predicted. | Title page |
| Abstract | 2 | D;V | Provide a summary of objectives, study design, setting, participants, sample size, predictors, outcome, statistical analysis, results, and conclusions. | 1-2 |
| **Introduction** | | | | |
| Background and objectives | 3a | D;V | Explain the medical context (including whether diagnostic or prognostic) and rationale for developing or validating the multivariable prediction model, including references to existing models. | 3-4 |
|  | 3b | D;V | Specify the objectives, including whether the study describes the development or validation of the model or both. | 4 |
| **Methods** | | | | |
| Source of data | 4a | D;V | Describe the study design or source of data (e.g., randomized trial, cohort, or registry data), separately for the development and validation data sets, if applicable. | 4-5 |
|  | 4b | D;V | Specify the key study dates, including start of accrual; end of accrual; and, if applicable, end of follow-up. | 4-5 |
| Participants | 5a | D;V | Specify key elements of the study setting (e.g., primary care, secondary care, general population) including number and location of centres. | 4-5 |
|  | 5b | D;V | Describe eligibility criteria for participants. | 4-5 |
|  | 5c | D;V | Give details of treatments received, if relevant. | NA |
| Outcome | 6a | D;V | Clearly define the outcome that is predicted by the prediction model, including how and when assessed. | 5/S1 |
|  | 6b | D;V | Report any actions to blind assessment of the outcome to be predicted. | NA |
| Predictors | 7a | D;V | Clearly define all predictors used in developing or validating the multivariable prediction model, including how and when they were measured. | 5-6, Table 1 |
|  | 7b | D;V | Report any actions to blind assessment of predictors for the outcome and other predictors. | NA |
| Sample size | 8 | D;V | Explain how the study size was arrived at. | Figure 1 |
| Missing data | 9 | D;V | Describe how missing data were handled (e.g., complete-case analysis, single imputation, multiple imputation) with details of any imputation method. | NA |
| Statistical analysis methods | 10a | D | Describe how predictors were handled in the analyses. | NA |
|  | 10b | D | Specify type of model, all model-building procedures (including any predictor selection), and method for internal validation. | NA |
|  | 10c | V | For validation, describe how the predictions were calculated. | Table 1 |
|  | 10d | D;V | Specify all measures used to assess model performance and, if relevant, to compare multiple models. | 10 |
|  | 10e | V | Describe any model updating (e.g., recalibration) arising from the validation, if done. | NA |
| Risk groups | 11 | D;V | Provide details on how risk groups were created, if done. | NA |
| Development vs. validation | 12 | V | For validation, identify any differences from the development data in setting, eligibility criteria, outcome, and predictors. | 7-8 |
| **Results** | | | | |
| Participants | 13a | D;V | Describe the flow of participants through the study, including the number of participants with and without the outcome and, if applicable, a summary of the follow-up time. A diagram may be helpful. | 7/ Figure 1 |
|  | 13b | D;V | Describe the characteristics of the participants (basic demographics, clinical features, available predictors), including the number of participants with missing data for predictors and outcome. | NA |
|  | 13c | V | For validation, show a comparison with the development data of the distribution of important variables (demographics, predictors and outcome). | Table 2 |
| Model development | 14a | D | Specify the number of participants and outcome events in each analysis. | NA |
|  | 14b | D | If done, report the unadjusted association between each candidate predictor and outcome. | NA |
| Model specification | 15a | D | Present the full prediction model to allow predictions for individuals (i.e., all regression coefficients, and model intercept or baseline survival at a given time point). | NA |
|  | 15b | D | Explain how to the use the prediction model. | NA |
| Model performance | 16 | D;V | Report performance measures (with CIs) for the prediction model. | 8 |
| Model-updating | 17 | V | If done, report the results from any model updating (i.e., model specification, model performance). | 8-9/, Table 1 |
| **Discussion** | | | | |
| Limitations | 18 | D;V | Discuss any limitations of the study (such as nonrepresentative sample, few events per predictor, missing data). | 11 |
| Interpretation | 19a | V | For validation, discuss the results with reference to performance in the development data, and any other validation data. | 9-10 |
|  | 19b | D;V | Give an overall interpretation of the results, considering objectives, limitations, results from similar studies, and other relevant evidence. | 10-12 |
| Implications | 20 | D;V | Discuss the potential clinical use of the model and implications for future research. | 11-12 |
| **Other information** | | | | |
| Supplementary information | 21 | D;V | Provide information about the availability of supplementary resources, such as study protocol, Web calculator, and data sets. | S1-S2 |
| Funding | 22 | D;V | Give the source of funding and the role of the funders for the present study. | 14 |

*Items relevant only to the development of a prediction model are denoted by D, items relating solely to a validation of a prediction model are denoted by V, and items relating to both are denoted D;V. We recommend using the TRIPOD Checklist in conjunction with the TRIPOD Explanation and Elaboration document.
